# Efficient Aperture Fill Time Correction for Wideband Sparse Array Using Improved Variable Fractional Delay Filters

**DOI:** 10.3390/s24134327

**Published:** 2024-07-03

**Authors:** Jie Gu, Min Xu, Wenjing Zhou, Mingwei Shen

**Affiliations:** 1 National Key Laboratory of Electromagnetic Space Security, Chengdu 610036, China; minxumin@163.com; 2 College of Information Science and Engineering, Hohai University, Nanjing 210098, China; 15637357907@163.com (W.Z.); smw_nuaa@hhu.edu.cn (M.S.)

**Keywords:** wideband sparse array, variable fractional delay (VFD), minimax criterion, ADMM algorithm

## Abstract

To solve the problem of aperture fill time (AFT) for wideband sparse arrays, variable fractional delay (VFD) FIR filters are applied to eliminate linear coupling between spatial and time domains. However, the large dimensions of the filter coefficient matrix result in high system complexity. To alleviate the computational burden of solving VFD filter coefficients, a novel multi–regultion minimax (MRMM) model utilizing the sparse representation technique has been presented. The error function is constrained by the introduction of L2–norm and L1–norm regularizations within the minimax criterion. The L2–norm effectively resolves the problems of overfitting and non–unique solutions that arise in the sparse optimization of traditional minimax (MM) models. Meanwhile, the use of multiple L1–norms enables the optimal design of the smallest sub–filter number and order of the VFD filter. To solve the established nonconvex model, an improved sequential–alternating direction method of multipliers (S–ADMM) algorithm for filter coefficients is proposed, which utilizes sequential alternation to iteratively update multiple soft–thresholding problems. The experimental results show that the optimized VFD filter reduces system complexity significantly and corrects AFT effectively in a wideband sparse array.

## 1. Introduction

A wideband sparse array has the advantages of improving spatial resolution, reducing hardware complexity, and improving target detection and recognition performance. However, the aperture fill time (AFT) phenomenon, inherent to wideband arrays, significantly diminishes the performance of beamforming. Variable fractional delay (VFD) filters involve the implementation of precise variable delay in digital systems and are widely used for aperture correction in wideband arrays [1,2]. High–precision delay filters are usually represented as a sum of integer and fractional terms. While cascaded registers allow for the implementation of integer terms, fractional terms require more sophisticated techniques [3,4]. The pioneering Farrow structure [5], known for its flexible and efficient delay correction, modifies and extends the traditional structure, effectively reducing implementation complexity and improving design accuracy.

In general, the design of a Farrow structure VFD (FS–VFD) filter primarily involves the minimax method [6,7] and the weighted least squares (WLS) method [8,9]. The minimax method is implemented by minimizing the maximum magnitude value between the actual frequency response and the ideal frequency response, ensuring accuracy within the approximation error range [7]. Some studies have transformed the solution problem into convex optimization problems in order to ensure the optimal value of the coefficient solution; it still requires numerous iterations and updates, leading to high computational complexity. The WLS design is implemented by minimizing the mean square error of the two frequencies, yielding an optimal closed solution [8]. However, the higher–order filter design leads to a cascading increase in computational complexity for both design criterion, necessitating additional storage resources [9]. High computational complexity and unstable values affect the efficiency of FS–VFD filters, so an ideal design approach needs to ensure accuracy while reducing computational complexity.

FS–VFD FIR filters comprise several sub–filters with a large number of coefficients, distinguishing them from the numerical scalars in digital FIR filters [10]. To reduce computation, the literature [11] reduces the number of filter coefficients using the WLS design criterion and the (inverse) symmetry of the coefficients but does not take into account the change in system complexity. Filter coefficient sparse processing is an optimization technique aimed at reducing system complexity and improving processing efficiency by reducing the number of non–zero coefficients in a filter [12,13]. Through sparse optimization, we can obtain a more compact and efficient filter structure, which is especially important for resource–constrained environments or application scenarios requiring real–time performance. The literature [14] studies VFDs with sparse coefficients for reducing computation. Leveraging the polynomial properties of the coefficients, a two–stage technique is proposed for a VFD FIR filter, aimed at obtaining a least squares solution under sparse target coefficients. To reduce system complexity and computation of the Farrow structure, the literature [15] considers sparse–optimized Farrow structure VFD (SFS–VFD) filters based on least squares and coefficient (inverse) symmetry to ensure high accuracy and efficient correction of aperture effects. It is noteworthy that while much research focuses on implementing FS–VFD filters with the WLS criterion, there remains a gap in related research based on the minimax criterion [16].

Therefore, this paper proposes a new multi–regulation minimax sparse model for VFD design. The error function is constrained by introducing *L*2–norm and *L*1–norm regularizations under the minimax criterion. The *L*2–norm solves the overfitting and non–unique solution problems of the traditional MM model in sparse optimization, and the *L*1–norm achieves the optimal design of the smallest sub–filter number and order for VFD filters. An improved sequential ADMM algorithm is proposed for solving this nonconvex model. Subsequently, the proposed MRMM–VFD filters are applied to a wideband sparse array to correct the AFT. Finally, simulation results verify the effectiveness and feasibility of MRMM–VFD filters.

## 2. Problem Formulation

**Figure 1:**
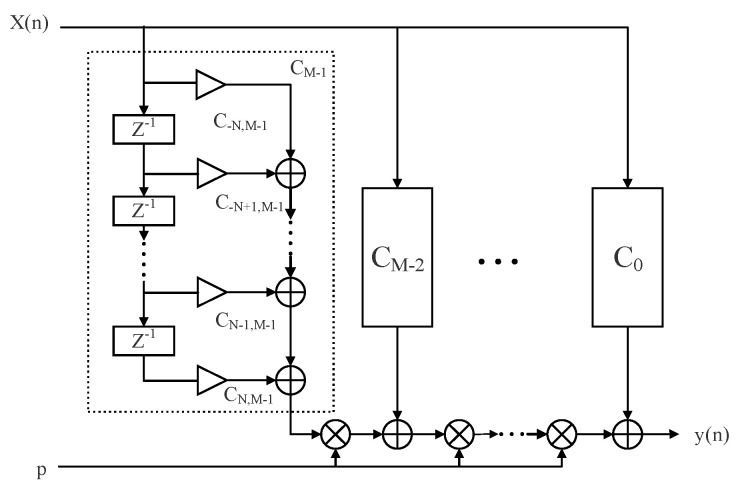
The Farrow structure the VFD FIR filter.

The ideal frequency response of a VFD filter is expressed as follows:

(1) $$H_{d}(\omega ,p)=e^{-j\omega p}\;$$

where ω∈[0,απ] is the passband range, and 0<α≤1, p∈[−0.5,0.5] is the fractional delay parameter range. The block diagram of the VFD filter structure based on the Farrow structure is shown in Figure 1. Therefore, the actual filter frequency response can be expressed as follows:

(2) $$H(\omega ,p)=\sum_{n=-N}^{N+1}\sum_{m=0}^{M}a(n,m)p^{m}e^{-j\omega n}$$

where a(n,m) are the filter coefficients; *M* is the number of sub–filters; and *N* is the sub–filter order. Since VFD filters have coefficient (anti)symmetry, taking an odd number *N* as an example, the coefficients of the even sub–filter (with symmetry) and the odd sub–filter (with anti–symmetry) are split to obtain the following form:

(3) $$H(\omega ,p)=\sum_{m=0}^{M_{e}}c_{m}^{T}b_{em}p^{2m}-j\sum_{m=1}^{M_{o}}s_{m}^{T}b_{om}p^{2m-1}$$

where

(4) $$\left\{\begin{array}{l} c_{m}^{T}=[\cos(\frac{1}{2}\omega )\cos(\frac{3}{2}\omega )\ldots {\cos((N+\frac{1}{2})\omega )]}^{t}\\ s_{m}^{T}=[\sin(\frac{1}{2}\omega )\;\sin(\frac{3}{2}\omega )\ldots {\sin((N+\frac{1}{2})\omega )]}^{t} \end{array}\right.$$

Convert it into the following form:

(5) $$H(\omega ,p)=f^{T}b_{e}-jg^{T}b_{o}$$

where

(6) $$f_{m}^{T}=c_{m}^{T}p^{2m},g_{m}^{T}=s_{m}^{T}p^{2m-1}$$

(7) $$\left\{\begin{array}{l} f^{T}=\left[f_{0}^{T}f_{1}^{T}\;\cdots \;f_{I}^{T}\right]\\ g^{T}=\left[g_{1}^{T}g_{2}^{T}\;\cdots \;g_{I+1}^{T}\right] \end{array}\right.$$

The minimax design of a VFD filter refers to minimizing the peak error between the ideal frequency response and the actual frequency response. The solution problem for the filter coefficients of the minimax model is given as follows:

(8) $$\mathrm{minimize}\;\max|H(\omega ,p)-H_{d}(\omega ,p)|$$

The error function is defined as follows:

(9) $$e_{H}(\omega ,p)=H(\omega ,p)-H_{d}(\omega ,p)=e_{R}(\omega ,p)-je_{I}(\omega ,p)$$

where

(10) $$\left\{\begin{array}{l} e_{R}(\omega ,p)=fb_{e}-\cos(\omega p)\\ e_{I}(\omega ,p)=gb_{o}-\sin(\omega p) \end{array}\right.$$

Then, problem (8) can be transformed into the following form:

(11) $$\mathrm{minimize}\;{\left\|fb_{e}-\cos(\omega p)+j(gb_{o}-\sin(\omega p))\right\|}_{\infty }$$

where $||\;\cdot {\;||}_{\infty }$ denotes the *L*_∞_ norm.

## 3. Improved Sparse–Constrained Design for the VFD FIR Filter

### 3.1. The Cost Function Based on the Multi–Regulation Minimax Criterion

Building upon the traditional MM model established in the above section, SDP and SOCP algorithms are usually used to optimize the solution [17] but can easily lead to high system complexity. Owing to the characteristics of VFD filters with more near–zero coefficients, sparse constraint theory is introduced into the optimization of filter weight coefficients, and an improved MM model, the multi–regulation minimax (MRMM) model, is proposed in this paper. The model is introduced with *L*2 and *L*1 regularization to constrain the model coefficients. The *L*2–norm solves the overfitting and non–unique solution problems of the traditional MM model in sparse optimization [18]. The *L*1–norm realizes the design of the minimum number of sub–filters and orders of the VFD filter. The MRMM model is formulated as follows:

(12) $$\mathrm{minimize}{\left\|\Phi x-\psi \right\|}_{\infty }+{\left\|y\right\|}_{2}^{2}+\lambda_{1}\sum_{n=0}^{(N+1)(M+1)-1}{\left\|\sum_{k=0}^{n}x_{(N+1)(M+1)-k}\right\|}_{1}+\lambda_{2}\sum_{n=1}^{N+1}\sum_{m=0}^{M}{\left\|\sum_{k=1}^{n}x_{\left(N+1-k)+M(N+1\right)}\right\|}_{1}$$

where the filter coefficient vector $x=[b_{e},b_{o}{]}^{T}$ and the parameters Φ and ψ in the term $||\;\cdot {\;||}_{\infty }$ are related to Equation (11) in Section 2. According to reference [19], the diagonal elements of H are associated with the symmetry of the coefficients of the filter. y is the vector associated with the real impulse response a(n,m) of the filter. According to coefficient symmetry b(n,m)=2a(n,m), for m=0,1,⋯,M, we define that the diagonal elements of H are $h_{1}=h_{2}=\ldots =h_{\left(N+1)(M+1\right)}=1/2$, and the diagonal matrix H can be reduced to a multiple of the unit matrix $I_{\left(N+1)(M+1\right)}$. In the *L*_∞_ term, we give

(13) $$\Phi =\left[\begin{array}{cc} f & 0\\ 0 & g \end{array}\right],\;\psi =\left[\begin{array}{c} \cos(\omega p)\\ \sin(\omega p) \end{array}\right]$$

The first and second terms in problem (12) denote the minimax error of the VFD filter; the third term denotes the optimal filter order constraint; and the fourth term denotes the optimal number of sub–filters constraint. The last two sparse terms should essentially be formulated as *L*0–norm problems. However, they cannot be solved directly due to NP Hard and a highly nonconvex nature. Therefore, the last two sparse terms are converted to *L*1–norm form after relaxation [20,21]. It can be seen that the orders of all sub–filters are optimized simultaneously by the second *L*1–norm term, which has a complex nested summation process and cannot be solved directly. Based on the properties of paradigm triangular inequality $||\sum_{i}b_{i}||\leq \sum_{i}\left||b_{i}|\right|$ [22], problem (12) can be approximated as follows:

(14) $$\mathrm{minimize}{\left\|\Phi x-\psi \right\|}_{\infty }+x^{T}Hx+\lambda_{1}\sum_{n=0}^{(N+1)(M+1)-1}\sum_{k=0}^{n}\left|x_{(N+1)(M+1)-k}\right|+\lambda_{2}\sum_{n=1}^{N+1}\sum_{k=1}^{n}\left[\left|x_{\left(N+1-k\right)}\right|+\ldots +\left|x_{\left((N+1-k)+M(N+1)\right)}\right|\right]$$

A further derivation gives the following:

(15) $$\mathrm{minimize}{\left\|\Phi x-\psi \right\|}_{\infty }+x^{T}Hx+\lambda_{1}\sum_{n=0}^{(N+1)(M+1)-1}((N+1)(M+1)-n)\left|x_{(N+1)(M+1)-n}\right|+\lambda_{2}\sum_{n=1}^{\left(M+1)(N+1\right)}rem[\frac{n}{\left(N+1\right)}]\left|x_{n}\right|$$

The last two items are integrated and reformulated in *L*1–norm form as follows:

(16) $$\mathrm{minimize}{\left\|\Phi x-\psi \right\|}_{\infty }+x^{T}Hx+\lambda_{1}{\left\|Qx\right\|}_{1}+\lambda_{2}{\left\|Sx\right\|}_{1}$$

*Q* and *S* in the formula are, respectively, as follows:

(17) $$\left\{\begin{array}{l} Q=diag(1,2,\ldots ,(N+1)(M+1))\\ s_{i}=diag(1,2,\ldots ,N+1)\\ S=diag(s_{1},s_{2},\ldots ,s_{M+1}) \end{array}\right.$$

### 3.2. S–ADMM Algorithm for Computing VFD Filter Coefficients

Since problem (16) needs to solve the optimization problem of the minimum sum of different paradigms, and the solution variables are multi–dimensional and complex, it is difficult for general optimization algorithms or tools to find the optimal solution. The ADMM algorithm combines the decomposability of the dual ascent method with the superior convergence of the multiplier method, making it suitable for solving large–scale distributed optimization problems.

The core idea of the ADMM algorithm is to decompose the original high–dimensional optimization problem into several low–dimensional subproblems, and gradually approach the optimal solution by solving these subproblems with alternating iterations. This decomposition and iteration strategy enables the algorithm to effectively deal with high–dimensional data and has better convergence and solving efficiency. However, the standard ADMM algorithm is only applicable to the problem of minimizing the sum of two norms. Therefore, in order to solve problem (3), the ADMM algorithm was improved and a sequential ADMM algorithm is proposed.

By introducing the auxiliary variables $z_{1},z_{2},z_{3}$, problem (16) is expressed as a minimum optimization problem with constraints as follows:

(18) $$\begin{array}{l} \mathrm{minimize}\;{\left\|z_{1}-\psi \right\|}_{\infty }+\frac{1}{2}x^{T}Hx+\lambda_{1}{\left\|z_{2}\right\|}_{1}+\lambda_{2}{\left\|z_{3}\right\|}_{1}\\ subject\;to\;\left[\begin{array}{l} \Phi \\ Q\\ S \end{array}\right]x+\left[\begin{array}{l} -I\\ 0\\ 0 \end{array}\right]z_{1}+\left[\begin{array}{l} 0\\ -I\\ 0 \end{array}\right]z_{2}+\left[\begin{array}{l} 0\\ 0\\ -I \end{array}\right]z_{3}=0 \end{array}$$

The variable *x* of the original problem is decomposed by the S–ADMM algorithm into multiple variables for sequential alternating iterations. Through constructing the augmented Lagrange function and updating the independent variables and the Lagrange multipliers, the iterations are repeated and stopped until the residuals reach a predetermined accuracy, thereby determining the optimal values of the variables. By defining the Lagrange multiplier vector $u=[u_{1},u_{2},u_{3}]$, the following iterative solution formula is obtained using the dual gradient descent method:

(19) $$\left\{\begin{array}{l} {\bar{x}}^{k+1}=\underset{x}{\arg\min}L_{\rho }(x,z_{1}^{k},z_{2}^{k},z_{3}^{k},u^{k})\\ =\underset{x}{\arg\min}\;\frac{1}{2}x^{T}Hx+\frac{\rho_{1}}{2}{\left\|\Phi x-\psi -z_{1}^{k}+u_{1}^{k}\right\|}_{2}^{2}+\frac{\rho_{2}}{2}{\left\|Qx-z_{2}^{k}+u_{2}^{k}\right\|}_{2}^{2}+\frac{\rho_{3}}{2}{\left\|Sx-z_{3}^{k}+u_{3}^{k}\right\|}_{2}^{2}\\ x^{k+1}=\beta {\bar{x}}^{k}+(1-\beta x^{k})\\ z_{1}^{k+1}=\underset{z_{1}}{\arg\min}L_{\rho }(x^{k},z_{1},u_{1}^{k})=\underset{z_{1}}{\arg\min}\;{\left\|z_{1}-\psi \right\|}_{\infty }+\frac{\rho_{1}}{2}{\left\|\Phi x^{k}-z_{1}+u_{1}^{k}\right\|}_{2}^{2}\\ z_{2}^{k+1}=\underset{z_{2}}{\arg\min}L_{\rho }(x^{k},z_{2},u_{2}^{k})=\underset{x}{\arg\min}\;\lambda_{1}{\left\|z_{2}\right\|}_{1}+\frac{\rho_{2}}{2}{\left\|Qx^{k}-z_{2}+u_{2}^{k}\right\|}_{2}^{2}\\ z_{3}^{k+1}=\underset{z_{3}}{\arg\min}L_{\rho }(x^{k},z_{3},u_{3}^{k})=\underset{x}{\arg\min}\;\lambda_{2}{\left\|z_{3}\right\|}_{1}+\frac{\rho_{3}}{2}{\left\|Sx^{k}-z_{3}+u_{3}^{k}\right\|}_{2}^{2} \end{array}\right.$$

where β takes the value in the range of (0, 1]. It is the core correction parameter that ensures the convergence of the algorithm [23]. It is not difficult to find that solving for *x* is a quadratic optimization problem, while solving for the auxiliary variable $z_{1},z_{2},z_{3}$ is a multi–soft–threshold optimization problem. Multiple variables are iteratively updated in an alternating sequence and constraints are taken into account using the Lagrange multiplier *u*. The iterative formula for the Lagrange multiplier vector u is as follows:

(20) $$\left\{\begin{array}{l} u_{1}^{k+1}=u_{1}^{k}+\rho_{1}(\Phi x^{k}-z_{1}^{k+1})\\ u_{2}^{k+1}=u_{2}^{k}+\rho_{2}(Qx^{k}-z_{2}^{k+1})\\ u_{3}^{k+1}=u_{3}^{k}+\rho_{3}(Sx^{k}-z_{3}^{k+1}) \end{array}\right.$$

The algorithm sets a predefined termination condition $\frac{{\left\|x_{k+1}-x_{k}\right\|}_{2}}{\left(N+1)(M+1\right)}\leq \mu$ and determines whether the convergence criterion is met, where μ is the threshold value. If it is satisfied, the iteration is stopped; the optimal solution is found; and the final coefficients are obtained by using the symmetry of the coefficients; otherwise, the iteration is continued.

The coefficient vector *x*, i.e., the corresponding even sub–filter coefficients $b_{e}$ and odd sub–filter coefficients $b_{o}$, is computed according to the S–ADMM algorithm. The complete filter coefficient matrix is obtained using the following coefficient (inverse) symmetry formula:

(21) b(n,m)=2a(n,m), for m=0, 1,⋯, M

## 4. Wideband Sparse Array AFT Correction Using MRMM–VFD

Sparse array design strives to optimally deploy sensors to achieve desirable beamforming characteristics, reduce system hardware costs, and lower computational complexity. In wideband signal models, sparse arrays can avoid conflicts caused by frequency spread of the signal. Reference [24] frames the design problem as optimally selecting *P* sensors out of *N* possible equally spaced locations (*P* << *N*), and a wideband sparse array design method based on the tapped delay line (TDL) structure was proposed.

Assume that all of the sensors are omnidirectional with the same response, and the signals impinge upon the array from the far field. The beamformer output y[n] is a linear combination of differently delayed versions of the received array signals $x_{m}[n]$, m=0,⋯,M−1. The distance from the zeroth sensor to the subsequent sensor is denoted by $d_{m}$ for m=0,⋯,M−1, with $d_{0}=0$. And the incident signal arrives at an angle θ.

The steering vector of the array as a function of the normalized frequency $\Omega =wT_{s}$ and the arrival angle θ is as follows:

(22) $$\mathrm{minimize}{\left\|\Phi x-\psi \right\|}_{\infty }+x^{T}Hx+\lambda_{1}\sum_{n=0}^{(N+1)(M+1)-1}\sum_{k=0}^{n}\left|x_{(N+1)(M+1)-k}\right|+\lambda_{2}\sum_{n=1}^{N+1}\sum_{k=1}^{n}\left[\left|x_{\left(N+1-k\right)}\right|+\ldots +\left|x_{\left((N+1-k)+M(N+1)\right)}\right|\right]$$

where $\mu_{m}=\frac{d_{m}}{cT_{s}}$ for m=0, 1,⋯, M−1, and ${\left\{\cdot \right\}}^{T}$ indicates a transpose operation. The response of the array is then given by the following:

(23) $$P(\Omega ,\theta )=w^{H}s(\Omega ,\theta )$$

where $w^{H}$ is the Hermitian transpose of the weight vector of the array, which is given by the following:

(24) $$w={\left[\begin{array}{llll} w_{0}^{T} & w_{1}^{T} & \ldots & w_{M-1}^{T} \end{array}\right]}^{T}$$

(25) $$w_{m}={\left[\begin{array}{llll} w_{m,0} & w_{m,1} & \ldots & w_{m,J-1} \end{array}\right]}^{T}$$

**Figure 2:**
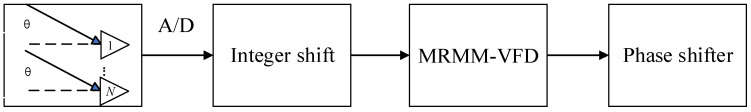
Aperture crossing correction of wideband sparse array.

Then, a compressive sensing sparse algorithm is used to match the array response with the desired/reference response, resulting in a sparse wideband array distribution [24]. For the obtained wideband sparse array, the MRMM–VFD filter is employed for aperture crossing correction, with the specific operational diagram shown in Figure 2.

The MRMM–VFD filter proposed in this paper has coefficient invariance, indicating that only the delay input needs to be altered while the filter coefficients remain unchanged when processing with different time delay parameters. In the aperture fill time correction of wideband sparse arrays, the MRMM–VFD greatly reduces computational complexity and system complexity.

## 5. Experimental Analysis

To verify the effectiveness of the designed MRMM sparse algorithm, the error results for different parameter settings are given in this section. Regarding the filter order *N*, a higher value results in a steeper frequency response of the filter, allowing for more accurate delay performance. However, excessively high orders may induce system instability, leading to increased noise and distortion; here, the value of *N* was chosen to be 30. The number of sub–filters *M* was set to 5, 7, 9, and 11. Regarding the value of the number of sub–filters, the amplitude response error decreases with an increase in the number of sub–filters. However, when it increases to a certain amount, the error reduction is no longer obvious. Additionally, an excessively large number of sub–filters can lead to unnecessary group phase delay errors [11,25]. The cutoff frequency parameter α=1, i.e., the full–pass bandwidth range was chosen for the experiment. In the MRMM algorithm, the penalty factors $\lambda_{1}$ and $\lambda_{2}$ are two important regularization parameters, which control the trade–off between data fitting and sparsity. We kept adjusting these two parameters with a sampling interval of 0.01, and the designed MRMM–VFD can achieve better performance when $\lambda_{1}=0.11$ and $\lambda_{2}=0.27$. To evaluate the error accuracy of the filter, the maximum magnitude error, the normalized mean square error (NMSE), and the maximum group delay error were used as evaluation metrics [25], and the expressions are shown, respectively, as follows:

(26) $$\varepsilon_{1}=\max\{20\log\left|H(\omega ,p)-H_{d}(\omega ,p)\right|\}$$

(27) $$\varepsilon_{2}={\left(\frac{\int_{0}^{\alpha \pi }\int_{-0.5}^{0.5}|H(\omega ,p)-H_{d}(\omega ,p){|}^{2}dpd\omega }{\int_{0}^{\alpha \pi }\int_{-0.5}^{0.5}|H_{d}(\omega ,p){|}^{2}dpd\omega }\right)}^{\frac{1}{2}}$$

(28) $$\varepsilon_{p}=\max\{|\tau (\omega ,p)-p|\}$$

**Figure 3:**
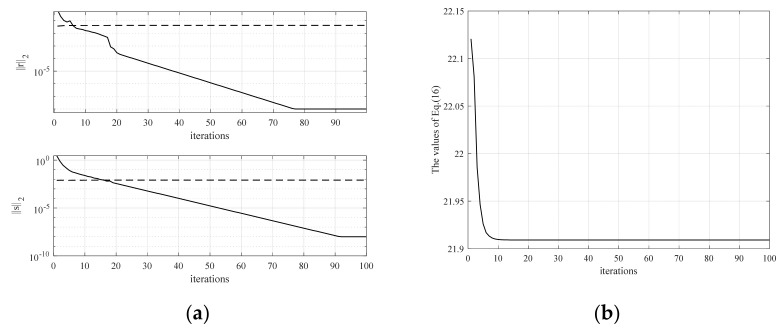
Convergence of the S−ADMM algorithm: (**a**) shows the convergence of ${\left\|r\right\|}_{2}$ and ${\left\|s\right\|}_{2}$; and (**b**) shows the convergence of the cost function given by Equation (16).

**Figure 4:**
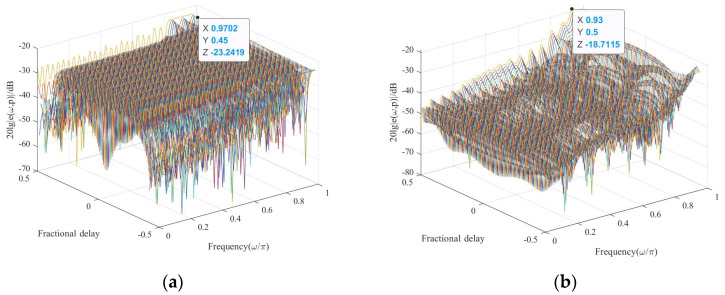

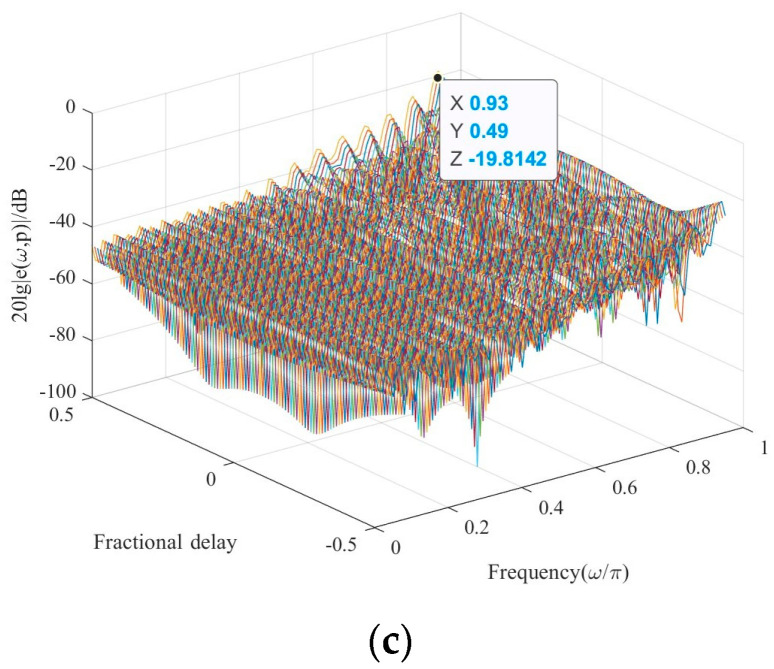
Amplitude–frequency response error plots for different algorithms. (**a**) is the MM algorithm; (**b**) is the S–WLS algorithm; and (**c**) is the MRMM algorithm.

Firstly, Figure 3a shows the convergence of primal and pairwise residuals in the S–ADMM algorithm, where the dotted lines are the set threshold; both of which can converge with an accuracy of 10^−5^. The algorithm sets the maximum number of iterations to 100 but as can be seen in Figure 3b, the minimum of function (16) is actually found after 20 iterations, with a clear and fast trend. The results of the standard MM algorithm and the weighted least squares sparse algorithm are compared to demonstrate the effectiveness of the MRMM sparse model in this paper, and the error results of different algorithms are given in Table 1. When *M* = 5, the maximum magnitude error and the normalized mean square error of the MRMM sparse algorithm are larger than the standard MM algorithm, but as the number of sub–filters increases, the maximum magnitude error gradually approaches the standard MM algorithm. The normalized mean square error of the MRMM algorithm is smaller compared to the S–WLS sparse algorithm for different numbers of sub–filters. In addition, the more the number of sub–filters, the smaller the filter error, but too many sub–filters will make the system redundant, so *M* = 9 is chosen for practical applications. The amplitude–frequency response error plots of the VFD filters using the MRMM algorithm, the standard MM algorithm, and the S–WLS sparse algorithm for the *N* = 30, *M* = 9 condition are given as shown in Figure 4. In comparison, the MRMM algorithm is flatter than the S–WLS sparse algorithm. The maximum magnitude error is −19.81 dB for the MRMM algorithm and −18.71 dB for the S–WLS sparse algorithm. The amplitude error is reduced by 1.0 dB compared to the un–sparse–processed MM algorithm. Therefore, it can be concluded that the proposed MRMM sparse algorithm is highly effective with high error accuracy.

**Figure 5:**
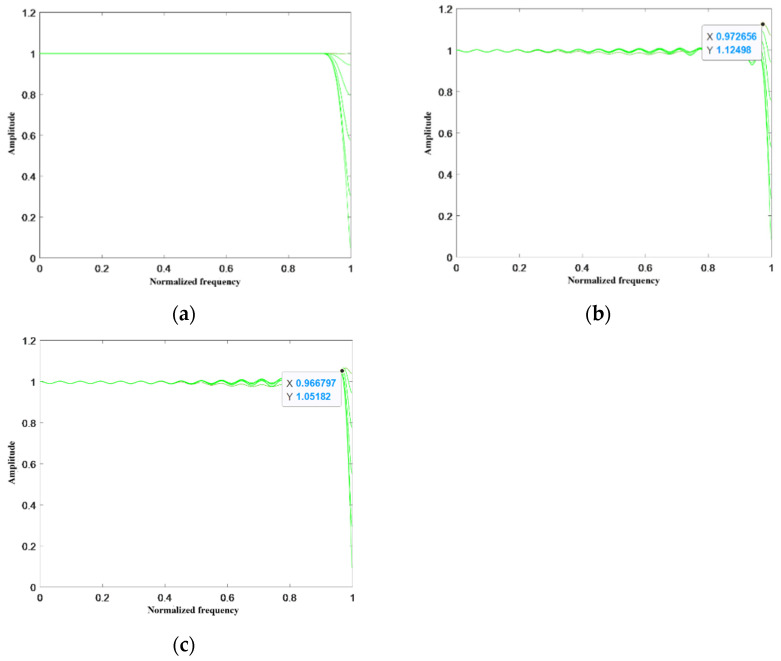
Amplitude–frequency response plots of different algorithms. (**a**) is the MM algorithm; (**b**) is the S–WLS algorithm; and (**c**) is the MRMM algorithm.

**Figure 6:**
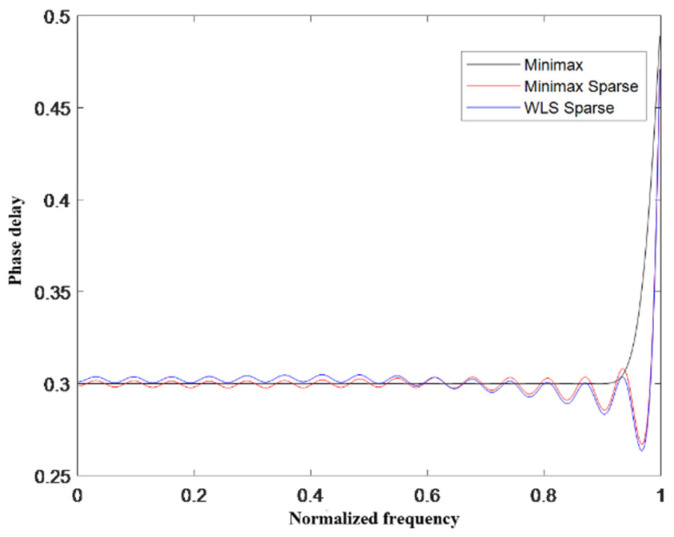
Phase–frequency plot for different algorithms at a fractional delay p=0.3.

Next, the amplitude–frequency response error and phase–frequency response error of the designed VFD filter were analyzed. Experimental results are given for the number of sub–filters *M* = 9 and the initial order of sub–filters *N* = 30. The magnitude–frequency response plots of different algorithms are given as shown in Figure 5, where it can be seen that the normalized magnitude of the un–sparse standard MM algorithm is close to one between the band range [0,0.9], and the normalized magnitude of the sparse–processed S–WLS algorithm and the MRMM algorithm fluctuates around one between the band range [0,0.9], but the range of fluctuation is within acceptable limits. In the bandwidth range [0.9,1], the maximum amplitude fluctuation of the MRMM algorithm is only 0.05, while the maximum amplitude fluctuation of the S–WLS algorithm in this range is 0.12. Next, Figure 6 shows the phase–frequency curves of the different algorithms where fractional delay *p* = 0.3; the maximum phase fluctuation of the S–WLS algorithm is 0.004; and the maximum phase fluctuation of the MRMM algorithm is only 0.002. Therefore, it can be proved that the MRMM algorithm proposed in this paper has more stable amplitude–frequency characteristics than the existing sparse algorithms.

To evaluate the sparsity of the MRMM algorithm and the effect of the sparse design on system complexity, the sparsity rate is defined as $\eta =\frac{P_{s}}{P}\times 100\%$ where *P*_s_ represents the number of zero coefficients after being sparse and P=2(N+1)(M+1) represents the number of filter coefficients under the initial setting. The experimental parameters are *M* = 9, *N* = 30, and *P* = 620. The results of the sparsity rate η and operation time Tc comparison of different algorithms are given in Table 2. The data in the table show that at *M* = 5 and *M* = 7, the sparsity rate of the MRMM algorithm is 66.67% and 75.40%, while the sparsity rate of the S–WLS algorithm is 69.89% and 75.80%, respectively. It can be seen that the sparsity of the S–WLS algorithm is a little bit better when the number of sub–filters is small, and the MRMM algorithm is better when the number of sub–filters is large. However, the sparsity of the sparse algorithms MRMM and S–WLS is much greater than that of the standard MM algorithm, regardless of the experimental conditions. In terms of computation time, we can see that the MRMM algorithm exhibits higher computational efficiency, especially when dealing with more sub–filters, and the MRMM algorithm is able to complete the computation faster. Therefore, it can be shown the sparsity of the MRMM algorithm designed in this paper fully satisfies our desired design.

Next, the system complexity of the VFD filter designed by the sparse MRMM algorithm is evaluated. The presence of zero coefficients at lower orders saves the use of corresponding multipliers, and the presence of zero coefficients at higher orders saves the corresponding multipliers and adders. By statistically analyzing the coefficient matrix elements, it can be determined that the filter designed by the standard MM algorithm requires 844 multipliers and 421 adders, which results in extremely high complexity for the system. However, the multipliers and adders required by the sparse MRMM algorithm are 240 and 119, corresponding to a reduction of 71.56% and 71.73%, respectively. It can be concluded that the use of multipliers and adders is significantly reduced by the MRMM sparse design algorithm, which effectively reduces system complexity.

**Figure 7:**
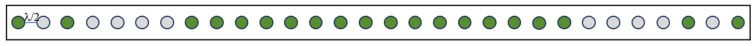
Sparse array location distribution.

The above experiment analysis proves that the improved VFD filter based on the MRMM model can achieve our purpose well. We then used it to correct the aperture effect in a wideband array. Assume that the angle of incidence of the signal is 10°; the center frequency is 1.2 GHz; and the $f_{H}:f_{L}=3:1$. The uniformly distributed array element spacing is $0.5\lambda_{\min}$, and the number of full arrays is 30. Then, the number of arrays after sparse reconstruction is 20, as shown in Figure 7, which is designed according to [24].

**Figure 8:**
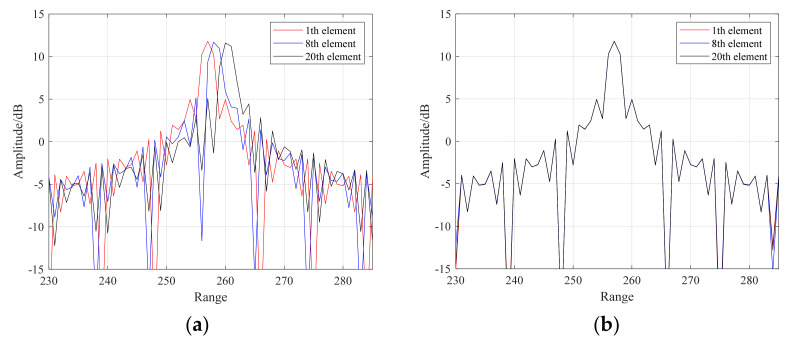
Wideband sparse array antenna orientation diagram. (**a**) is the result of aperture crossing; and (**b**) is the result of aperture correction.

**Figure 9:**
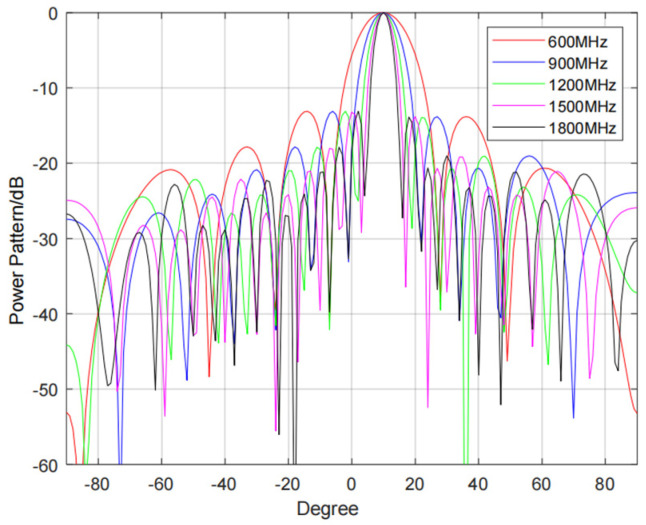
Wideband sparse array antenna orientation diagram.

Compared with the conventional wideband aperture effect correction for a TDL structure [24], our proposed MRMM–VFD–based wideband aperture effect correction method can keep the filter coefficients unchanged under different scan angles, and only the delay parameter needs to be adjusted. Compared with the LCMM–VFD filter, our proposed MRMM–VFD can significantly reduce the use of multipliers and adders and decrease system complexity. Figure 8a shows the aperture crossing phenomenon of a wideband sparse array, and it can be seen that serious pointing deviations occur in the received signals of different arrays. Figure 8b shows that the received signals of each array are aggregated to the same distance unit after aperture correction using the improved VFD filter, which completely solves beam dispersion. The antenna orientation diagrams of the wideband sparse array with different frequency signals are given in Figure 9. It can be seen above that it is effective to use the proposed VFD filter for wideband sparse arrays during aperture correction.

## 6. Conclusions

In order to reduce the system complexity of the VFD FIR filter, a new MRMM sparse algorithm under a great–minimal criterion is proposed. The error function is constrained by introducing *L*2 and *L*1 regularizations. To solve this nonconvex model, an improved S–ADMM algorithm is proposed, which uses sequential alternation to iteratively update multiple soft–threshold problems. Experimental results show that the proposed MRMM algorithm completely satisfies the desired error value and sparsity. The use of multipliers and adders is reduced by 71.56% and 71.73%, respectively, leading to a significant decrease in system complexity. Simulation results confirm that the MRMM–VFD effectively corrects AFT in a wideband sparse array.

## Figures and Tables

**Table 1:** Comparison of error performance of different algorithms.

	*M* = 5		*M* = 7		*M* = 9		*M* = 11	
	$\varepsilon_{1}$	$\varepsilon_{2}$	$\varepsilon_{1}$	$\varepsilon_{2}$	$\varepsilon_{1}$	$\varepsilon_{2}$	$\varepsilon_{1}$	$\varepsilon_{2}$
MM	−19.83	0.0067	−20.01	0.0061	−18.69	0.0085	−23.24	0.0047
S–WLS	−17.74	0.01	−20.78	0.0054	−18.71	0.0072	−20.44	0.0061
MRMM	−18.37	0.0097	−19.45	0.0080	−19.81	0.0066	−21.98	0.0058

**Table 2:** Comparison of sparsity and operation time of different algorithms.

	*M* = 5		*M* = 7		*M* = 9		*M* = 11	
	η	*Tc*	η	*Tc*	η	*Tc*	η	*Tc*
MRMM	66.67% (248)	0.16 s	75.40% (374)	0.29 s	82.64% (512)	0.61 s	85.75% (638)	0.73 s
LCMM	37.97% (139)	0.62 s	21.77% (108)	2.05 s	31.93% (198)	2.40 s	32.52% (242)	3.67 s
S–WLS	69.89% (260)	0.14 s	75.80% (376)	0.26 s	81.29% (504)	0.77 s	85.21% (634)	0.92 s

## Data Availability

The data used to support the findings of this study are available from the corresponding author upon request.
